# 可切除非小细胞肺癌新辅助免疫治疗的进展

**DOI:** 10.3779/j.issn.1009-3419.2020.103.07

**Published:** 2020-05-20

**Authors:** 帅博 王, 友生 毛

**Affiliations:** 100021 北京，国家癌症中心/国家肿瘤临床医学研究中心/中国医学科学院北京协和医学院肿瘤医院 Department of Thoracic Surgery, National Cancer Center/National Clinical Research Center for Cancer/Cancer Hospital, Chinese Academy of Medical Sciences and Peking Union Medical College, Beijing 100021, China

**Keywords:** 肺肿瘤, 新辅助治疗, 免疫治疗, Lung neoplasms, Neoadjuvent therapy, Immunotherapy

## Abstract

可切除非小细胞肺癌(non-small cell lung cancer, NSCLC)是一种潜在可治愈性疾病。尽管外科手术仍然是可切除NSCLC的主要治疗手段，但仍有部分患者术后出现局部复发和远距离转移。因此，为改善长期生存效果术前术后辅助治疗可能仍有必要。免疫检查点抑制剂已经临床试验证实其治疗效果，目前已被批准用于转移性NSCLC或部分Ⅲ期局部晚期NSCLC的一线或二线使用。免疫治疗在晚期肺癌的显著疗效使研究者开始关注免疫治疗是否用于可切除非小细胞肺癌中的新辅助治疗。本文对免疫治疗作为可切除非小细胞肺癌的新辅助治疗的研究进行综述。

## Abstract (en)

Resectable non-small cell lung cancer (NSCLC) is currently considered as a potentially curable disease. Surgery is still the main treatment mode for resectable NSCLC, but quite a few patients will have local recurrence and distant metastasis after surgery. Therefore, preoperative and postoperative adjuvant therapy may be necessary in order to improve the long term outcome. Immunocheckpoint inhibitor has been demonstrated clinically to be effective andapproved as first- or second-line treatment agent in metastatic NSCLC or partially locally advanced NSCLC. The remarkable efficacy of immunotherapy for advanced lung cancer has attracted more and more attention from the researchers to the role of immunotherapy as neoadjuvent therapy in resectable non-small cell lung cancer. This article systematically reviewed the clinical trials of neoadjuvant immunotherapy for resectable NSCLC before surgery.

肺癌是目前对人类健康和生命威胁最大的恶性肿瘤之一。据估计2019年在美国肺癌新发病例22.8万人，死亡14.3万人^[1]^。2019年国家癌症中心肿瘤登记办公室调查显示2015年我国新发肺癌病例约为78.7万例，发病率为57.26/10万。肺癌在我国发病率和死亡率均居所有癌症之首。其发病率在男性发病居首位，在女性中居第二位。2015年在我国因肺癌死亡共计63.1万例，死亡率为45.87/10万。肺癌死亡率在男性和女性均居恶性肿瘤死亡率的首位^[2]^。因此，我国肺癌防控形势非常严峻。

2013年世界肺癌大会及2014年欧洲肺癌大会均提出免疫治疗将开启肺癌治疗的新时代。最初的研究显示转移性非小细胞肺癌(non-small cell lung cancer, NSCLC)和黑色素瘤免疫治疗显著提高了患者的生存期。CA209-003研究显示已接受治疗的晚期NSCLC患者中应用nivolumab的2年总生存期(overall survival, OS)为23%-29%，5年总生存期为16%。针对不可切除的局部晚期NSCLC的治疗，近期公布的PACIFIC研究显示根治性放化疗后联合免疫巩固治疗后患者中位无进展生存期(progression-free survival, PFS)延长了11.2个月(16.8个月*vs* 5.6个月)，明显延长患者OS^[3, 4]^。

目前免疫检查点抑制剂已经成为进展期肺癌的重要治疗方法。在美国国立综合癌症网络(National Comprehensive Cancer Network, NCCN)指南中对于进展期程序性死亡受体-配体1 (programmed cell death-ligand 1, PD-L1)阳性表达(≥ 1%)、且表皮生长因子受体(epidermal growth factor receptor, *EGFR*)和间变性淋巴瘤激酶(anaplastic lymphoma kinase, *ALK*)、*ROS1* (C-ros oncogene 1 receptor tyrosine kinase)、*BRAF* (v-Raf murine sarcoma viral oncogene homolog B)基因表达阴性或未知的肺鳞癌，如对Pembrolizumab或Atezolizumab无禁忌证，一线方案首选卡铂+紫杉醇/白蛋白紫杉醇+ Pembrolizumab^[5]^。免疫治疗在不可切除肺癌中取得的良好疗效使研究者们逐步探索其在可切除NSCLC中的潜在作用，多项临床研究开始探索新的免疫治疗模式^[6, 7]^。

## 1 肺癌新辅助免疫治疗的合理性

新辅助治疗是指外科手术前的辅助治疗，包括新辅助化疗、新辅助放疗、新辅助免疫治疗以及三者的联合治疗等。

肿瘤免疫检查点抑制剂不同于手术、化疗和放疗，它是通过阻断T淋巴细胞与抗原提呈细胞之间抑制性信号通路，激活肿瘤特异性T细胞的抗肿瘤作用，从而实现抗肿瘤作用。其主要靶点有细胞毒性T淋巴细胞相关蛋白4 (cytotoxic T lymphocyte-associated antigen-4, CTLA-4)、PD-1/PD-L1、B/T淋巴细胞衰减因子(B and T-cell lymphocyte attenuator, BTLA)、T细胞活化的含V区免疫球蛋白抑制物(V-domain Ig suppressor of T-cell activation, VISTA)、TIM-3等^[8]^。

目前的研究结果显示，新辅助免疫治疗有一定合理性。手术前肿瘤内存在多数表达免疫检查点抑制剂靶标的细胞，进而在免疫治疗时大量的肿瘤抗原有助于激活大量肿瘤浸润淋巴细胞，引发持久的抗肿瘤效应。术前诱导的系统性免疫反应可使机体产生长期免疫记忆，预防肿瘤复发，而术后患者因肿瘤的切除无法产生免疫介导的持续的抗肿瘤效应^[9]^。

机体行免疫治疗后产生的免疫激活效应可以消灭肿瘤微转移。新辅助免疫治疗产生的抗肿瘤效应可以让肿瘤缩小。新辅助免疫治疗过程中准确的疗效评估能够快速指导后续治疗。新辅助免疫治疗可以在手术行淋巴结清扫前最大化激活机体的抗肿瘤效应。相比术后患者初治患者有更好地耐受性和更强的系统性抗肿瘤T细胞反应。新辅助免疫治疗可能增加其他治疗方式的抗肿瘤效应。新辅助免疫治疗期间的效果有潜在预测生存期的可能^[7]^。

在一项基于三阴性乳腺癌的临床前试验中，作者证实了在小鼠自发转移性乳腺癌的两种模型中，新辅助相较辅助免疫疗法可显著提高肿瘤特异性CD8^+^ T细胞，产生更强的抗肿瘤效应，抑制肿瘤微转移^[10]^。部分研究显示在早期NSCLC中PD-1通路的阻断可能通过降低肿瘤亚群的异质性、提高宿主免疫反应性来实现抗肿瘤效应^[11]^。在一项使用免疫重编程上调体内T细胞PD-1/PD-L1通路的研究中，模式小鼠产生了对体内早期NSCLC的持久性抗肿瘤效应，这一结果强调了在NSCLC进展的早期阶段激活PD-1/PD-L1通路的重要性^[12]^。

## 2 新辅助免疫治疗的临床研究

新辅助免疫治疗在可切除NSCLC的应用源于在Ⅰ期-Ⅲa期可手术切除患者中进行的小样本的随机对照研究^[13]^，目前正在探索的关于免疫治疗的治疗模式主要有：新辅助免疫治疗→手术治疗；新辅助免疫治疗→手术治疗→辅助免疫治疗；新辅助免疫+化疗→手术治疗→辅助免疫治疗等^[14]^ (表 1)。

**1:** 已有初步结果的临床试验 Resultful clinical trials

Abbreviation	Trial identifier	Start date	Phase	Neoadjuvant therapy	Stage	Adj. Therapy	Target Enroll	Enrollment now	Surgery	DR-AEs(≥ grade 3)	no-delay surgery rate	MPR	pCR
IO alone													
LCMC3	NCT02927301	2017	Ⅱ	Atezolizumab	Ⅰb; Ⅱ; Ⅲa; Selected Ⅲb	1 year	180	101	90	6(6%)	90 (89.1%)	18%	4% (6/82 PR, 72 SD, 4 PD)
MK3475-223	NCT02938624	2017	Ⅰ	Pembrolizumab	Ⅰ; Ⅱ	No	28	15	13	2 (13%)	13(87%)	40%	无
NEOSTAR	NCT03158129	2017	Ⅱ	Nivolumab+surgery *vs* Ipilimumab +Nivolumab+surgery	Ⅰ; Ⅱ; Ⅲa	No	N: 23 NI: 21	39	N: 4 (17%) NI: 2 (9.5%)	N: 22 (96%) NI: 17 (81%)	N: 22 (96%) NI: 17 (81%)	N: 4 (17%) NI: 7 (33%)	N: 2 (9%) NI: 4 (21%)
Cleckmate 159	NCT02259621	2016	Ⅱ	Nivolumab	Ⅰb; Ⅱ; Ⅲa	No	30	22	21	5 (23%)	20/21 (95%)	45%	无
ChiCTR-OIC-17013726		2017	Ⅰ	Sintilimab	Ⅰb; Ⅱ; Ⅲa	No	30	40	37	2 (5%)	30 (82.5%)	15 (40.5%)	6 (16.5%)
IO+													
	NCT02716038	2016	Ⅱ	MPDL3280A+Carboplatin+Nabpaclitaxel	Ⅰb; Ⅱ; Ⅲa	No	30	14	11	12 (85.7%)	11 (64%)	50.00%	21.40%
NADIM	NCT03081689	2017	Ⅱ	Nivolumab+Paclitaxel +Carboplatin	Ⅲa	1 year	46	46	41	6/46	41 (100%)	35 (85.4%)	25 (71.4%)
PD: progressive disease; SD: stable disease; PR: partial response; CR: complete esponse.													

### 2.1 新辅助免疫治疗+手术治疗

#### 2.1.1 CheckMate-159

2018年*N Eew J Med*发表了成人可切除NSCLC (Ⅰ期-Ⅲa期)术前应用Nivolumab的初步结果。研究共纳入了21例接受新辅助Nivolumab 2个周期治疗(3 mg/kg)的患者，研究结果显示有20例患者达到了完全切除，在20例接受完全手术切除的患者中有9例(45%)达到了主要病理学缓解(major pathological response, MPR)^[13]^。随后在2019年美国临床肿瘤学会年会上Forde报道了研究患者的随访结果，在中位随访30个月时，20例患者中有15例无病且存活。有2例患者死亡。24个月的中位无病生存率(disease-free survival, DFS)为69% (95%CI: 51%-93%)。

研究中共5例患者出现免疫治疗相关的副反应，除1例发生了3级免疫不良反应外，其余患者均未出现明显不良反应。值得注意的是研究人群未出现免疫治疗导致手术延迟。

研究对新辅助免疫治疗后的围手术期报告进行了分析，21例患者除1例患者(因疾病进展)未手术外均接受了手术治疗的患者中，14例患者行开胸肺切除术，3例患者行胸腔镜肺切除术，3例患者行机器人辅助下肺切除术。值得注意的是多数患者接受了开胸肺切除术而非胸腔镜下肺组织切除术，研究显示其原因是57% (12例)的患者分期较晚(Ⅱb期-Ⅲa期)，患者叶间裂、肺组织与胸壁、淋巴结与周围组织间存在粘连。心律失常(6例)是最常见的术后并发症。值得注意的是9例获得MPR的患者中，开胸手术仅占3例。新辅助免疫治疗对手术的影响需要进一步研究观察^[15]^。

该研究结果显示出现MPR的患者治疗后复发和转移较低。研究显示无论是否表达PD-L1患者均可获得主要病理缓解MPR。研究者同时分析了纳入患者的TMB情况，结果显示肿瘤突变负荷与患者达到MPR存在正相关^[16]^。

#### 2.1.2 NEOSTAR研究

旨在评价双联免疫治疗(Nivolumab联合Ipilimumab)与单药免疫治疗(Nivolumab)在新辅助治疗NSCLC中的疗效。这项Ⅱ期临床试验共入组44例患者，患者在手术前随机分配至两组，其中双联免疫治疗组共计21例，单药组共计23例。共接受3个周期治疗。研究根据第七版TNM分期对入组患者进行随机化分组，其中单药Nivolumab组分期为Ⅰa期4例(17%)、Ⅰb期7例(30%)、Ⅱa期2例(9%)、Ⅱb期5例(22%)、Ⅲa期5例(22%)。双联免疫治疗组分期为Ⅰa期4例(19%)、Ⅰb期8例(38%)、Ⅱa期5例(24%)、Ⅲa期4例(19%)。

在总人群中，双药Nivolumab+Ipilimumab组主要病理缓解率(MPR)与PCR共计达33%，优于单药Nivolumab组的17%。在接受手术的39例患者中，单药组共计22例，双药Nivolumab+Ipilimumab组共计17例。双药Nivolumab+Ipilimumab组主要病理缓解率(major pathological response, MPR)与PCR共计达44%，优于单药Nivolumab组的19%；在达到MPR的患者中影像学RECIST评估完全缓解(complete response, CR)加部分缓解(partial response, PR)的比例为60%。该研究中新辅助后顺利手术的患者为39例(9%)，5例(11%)患者新辅助治疗后未接受手术。

未接受手术的5例患者中，单药Nivolumab组及联合组各有1例患者未按照研究计划接受了手术治疗而出组，其余3例患者因严重副作用未能完成新辅助治疗。其中单药Nivolumab组1例(4%)，因1个周期治疗后发生3级缺氧，双联免疫治疗组2例(10%)，其中1例因1个周期治疗后发生3级腹泻，另1例因2个周期后发生2级肺炎^[17, 18]^。

研究同时报道了其手术结果，患者完成新辅助免疫治疗后行手术切除的中位间隔时间为31 d。8例(22%)患者因治疗相关副反应导致手术延期超过42 d，其中单药Nivolumab组3例，双联免疫治疗组5例。中位手术时间147 min，中位术中出血量100 mL，中位术后住院日4 d，无术中输血。约19%的患者手术时间长于4 h。27例患者(73%)接受了开胸肺切除术，7例(19%)接受了胸腔镜肺切除术，3例(8%)患者行机器人辅助肺切除术。手术难度的主观评价显示40%的外科医生认为新辅助免疫治疗术后的手术难度增大。术后并发症包括2例(5.1%)支气管内胸膜瘘和8例(20.5%)气胸。3级-5级治疗相关的副反应共计6例(13.6%)(单药组4例，联合组2例)^[19]^。

该研究显示获得MPR的患者治疗前肿瘤PD-L1表达较高，且在PD-L1 > 1%的患者中治疗后残余肿瘤更少，进而得出治疗前PD-L1较高的患者可能获得更大的抗肿瘤效应。研究还显示Nivolumab+Ipilimumab组新辅助治疗可诱导更多CD3^+^浸润性T细胞，T细胞多样性和记忆性T细胞也显著增加。

NEOSTAR研究对双联免疫治疗与单药免疫治疗新辅助治疗可切除NSCLC的疗效进行了探索，研究结论显示治疗有效性上双联免疫治疗更优，但双联免疫治疗面临的副反应可能会导致手术延期。

#### 2.1.3 MK3475-223研究

MK3475-223研究是一项对Ⅰ期-Ⅱ期NSCLC开展的新辅助Pembrolizumab的Ⅰ期、单臂、剂量递增和扩展队列试验。2019年美国临床肿瘤协会(American Society of Clinical Oncology, ASCO)上更新的结果显示共计15例患者接受了新辅助治疗，13例患者进行了手术。从免疫治疗结束至接受手术治疗的时间为30 d-45 d。在10例接受2个周期Pembrolizumab治疗的患者中4例患者(40%, 95%CI: 16.7%-68.8%)获得MPR。该研究的中位无病生存期(disease-free survival, DFS)时间为22.4个月。

虽然所有患者均未出现剂量限制性毒性(doses-schedule limiting toxicities, DLT)，但该研究中共计有2例(13%)因免疫治疗副反应导致的手术延迟及3例(20%)须接受激素治疗的3级以上治疗相关的副反应。4例(27%)手术相关的副反应及5例(33%)非手术相关的副反应。

MK3475-223研究并未观察到PD-L1表达与MPR之间的相关关系，提示早期NSCLC患者获得MPR的预测标志物可能与晚期疾病患者获得MPR的预测标志物有所差异。该研究分析了从最后一次Pembrolizumab治疗到接受手术治疗的不同间隔对于手术后MPR的影响。研究结果显示更长的治疗间隔可能对应了更高的MPR率。研究结果为可切除NSCLC的新辅助免疫治疗后选择手术的时机提供了一定的参考^[20]^。

#### 2.1.4 ChiCTR-OIC-17013726研究

ChiCTR-OIC-17013726研究是一项在中国开展，旨在评估为Sintilimab单药用于可切除NSCLC新辅助治疗的开放性、单中心、Ⅰb期研究。该研究共纳入40例(腺癌患者有驱动基因突变如*EGFR*、*ALK*者不入组) NSCLC患者(32例男性和8例女性)，其中鳞癌为33例(82.5%)，腺癌7例，共接受了2周期Sintilimab的治疗，随后37例患者在观察4周后进行了根治性切除。15例患者(40.5%, 95%CI: 30.2%-66.9%)达到了MPR，其中6例(16.2%, 95%CI: 6.2%-32.0%)具有完全病理缓解。

虽然共计18例(45%)患者经历了新辅助治疗相关的AE (treatment-related adverse events, TRAE)，但仅2例(5%)患者出现了3级-4级TRAE。

值得注意的该研究对新辅助治疗前后的所有患者均进行了PET-CT检查，在8例主病灶标准摄取值(standard uptake value, SUV)下降 > 30%的患者中，5例达到MPR (62.5%)。而在29例SUV下降≤ 30%或SUV升高的患者中，10例患者达到MPR (34.4%)。这一结果为筛选潜在受益患者提供了思路^[21-23]^。

### 2.2 新辅助免疫治疗+手术治疗+辅助免疫治疗

相对于CheckMate-159研究，LCMC3研究是一项大型多中心研究Ⅱ期临床研究，研究人群是可手术切除的NSCLC患者(Ⅰb期-Ⅲb期)。值得注意的是该研究纳入了部分Ⅲb期(T3N2、T4N2)的患者，纳入患者的T分期仅根据肿瘤大小决定，不纳入肿瘤浸润纵隔的患者。且入组患者未排除具有驱动基因突变(如*EGFR*、*ALK*)的患者。所有患者接受Atezolizumab治疗2个周期后接受手术。

该研究的中期的分析结果显示共计90例患者接受了手术治疗。除去8例驱动基因阳性患者，MPR率为15/82 (18%, 95%CI: 11%-28%)，4例(5%)患者达到完全病理缓解(pathological complete response, pCR)。但在8例驱动基因阳性(1例*ALK*阳性，7例*EGFR*阳性)患者中MPR率为3/8 (40%-50%)。

研究中29例患者出现3级-4级不良事件，其中6例出现了与治疗相关的副反应，2例出现非治疗相关的5级不良事件。但LCMC3研究结果显示PD-L1的表达与获得MPR无相关性；获得MPR和未获得MPR患者的TMB也无明显差异。提示病理缓解的疗效与PD-L1表达水平、TMB高低无相关性。新辅助免疫治疗后，获得MPR的患者出现NK细胞和粒细胞亚群的扩增、单核细胞亚群的减少^[16]^。

### 2.3 免疫治疗联合化疗的新辅助临床研究

#### 2.3.1 NADIM研究

NADIM是一项旨在探索免疫联合化疗在Ⅲa期NSCLC患者疗效的多中心临床研究。试验组在术前给予Nivolumab+紫杉醇+卡铂(3周)，值得注意的是手术在新辅助治疗后3周-4周进行，术后持续行Nivolumab治疗1年。截止到2019年5月该研究纳入了46例患者，共计41例患者接受手术。5例患者因尚未完成治疗计划还未手术。研究结果显示MPR为83%，pCR为71%。38例(93%)患者新辅助治疗后降期。RECIST的影像学评估显示PR率为71%，CR率为7%^[24]^。意向性治疗分析(intention to treatment, ITT)显示术后患者18个月的DFS为81% (95%CI: 61%-91%)，OS为91% (95%CI: 73-97%)。

这项研究的重要性在于研究中无论是病理学检查还是放射学检查标准，对新辅助化疗加免疫疗法的反应率都很高，新辅助治疗降期率也很高。但是两者评价标准不同且有差别，值得进一步研究。在该研究中接受免疫治疗+化疗的患者均及时接受了手术，未因疾病进展或毒性反应而提前退出研究。由于该项研究的启示，目前正在开展多项Ⅲ期临床试验(IMpower 030、KEYNOTE-671、CheckMate 816、CheckMate 77T)，这些研究将将进一步揭示新辅助免疫治疗+化疗模式的临床效果和未来在新辅助治疗中的地位^[25]^。

#### 2.3.2 NCT02716038

该研究是一项单臂Ⅱ期临床研究，应用卡铂加白蛋白紫杉醇和MPDL3280A (PD-L1抗体)进行术前新辅助治疗。2018年ASCO上公布了该研究的第一阶段结果，在纳入研究的14例患者中，Ⅲa期患者占85%，54%的患者PD-L1表达大于1%。最终11例患者成功进行手术切除，但该研究中12例(85.7%)患者出现3级-4级副反应(中性粒细胞减少)，9例(64.3%)患者因副反应接受了化疗剂量的减量治疗。1例患者术后出现与药物无关的并发症。7例(50%)患者达到MPR，3例(21.4%)患者达到pCR^[26]^。其结论显示PD-L1的表达与与MPR不相关。该研究的数据体现出新辅助免疫治疗+化疗模式的副反应带来的负面影响。研究提示无论PD-L1表达水平如何，患者均可从新辅助免疫治疗中获益。

总而言之，以上几项研究均提供了新辅助免疫检查点抑制剂在早期NSCLC中的初步应用结果。结果显示新辅助免疫治疗在可手术NSCLC患者中取得一定的疗效，尤其是新辅助免疫治疗加化疗效果更好，相对于单纯新辅助化疗，联合免疫治疗可取得更高的MPR及PCR率。但治疗副反应严重性不容忽视。免疫治疗开启了肺癌治疗的新时代，多种治疗模式如新辅助免疫治疗+化疗、新辅助免疫治疗+放疗等相继开展临床研究(表 2)。

**2:** 正在进行的临床试验 Ongoing clinical trials

Abbreviation	Trial identifier	Start date	Phase	Neoadjuvant therapy	Stage	Ajd. therapy	Target enroll	Primary outcomes	Secondary outcomes
IO alone									
NEOMUN	NCT03197467	2018	Ⅱ	Pembrolizumab	Ⅱ; Ⅲa	No	30	Safety; Path; RECIST	DFS; OS
IONESCO	NCT03030131	2017	Ⅱ	Durvalumab	Ⅰb; Ⅱ	No	81	Surgery resection R0	Path; Delay between surgery; AEs; DFS; OS; biomarkers; MPR
TOP 1501	NCT02818920	2017	Ⅱ	Pembrolizumab	Ⅰb; Ⅱ; Ⅲa	4 cycles	32	Surgical feasibility rate	ORR; DFS; Biomarker; TIL; CTC; Adverse events; Path
PRINCEPS	NCT02994576	2016	Ⅱ	Atezolizumab	Ⅰb; Ⅱ; Ⅲa	No	60	Major toxicities or morbidities	None
None	NCT03732664	2018	Ⅰ	Nivolumab	Ⅰ; Ⅱ; Ⅲa	3 cycles	40	Safety and Adverse effects	Rate of enrollment; Path; Radiographic Response
AEGEAN	NCT03800134	2019	Ⅲ	Durvalumab	Ⅱ; Ⅲ	No	300	mPR	pCR; OS; EFS; DFS
IO+ (without radiation)									
IMpower 030	NCT03456063	2018	Ⅲ	Atezolizumab+platinum-based chemotherapy *vs* Atezolizumab+platinum-based chemotherapy	Ⅱ; Ⅲa; selected Ⅲb	16 cycles	374	MPR; EFS	OS; OR; PCR; AEs
KEYNOTE-671	NCT03425643	2018	Ⅲ	Pembrolizumab	Ⅱ; Ⅲa; selected Ⅲb	13 cycles	786	EFS; OS	MPR; pCR; Aes
CheckMate 816	NCT02998528	2016	Ⅲ	Nivolumab+ipilimumab *vs* ipilimumab+platinum doublet *vs* platinum doublet	Ⅰb; Ⅱ; Ⅲa	No	350	EFS; PCR	OS; MPR; TTDM
MEDI4736	NCT02572843	2015	Ⅱ	Durvalumab	Ⅲa	2 cycles	68	EFS	EFS; OS; OR; PCR
NeoCOAST	NCT03794544	2019	Ⅱ	Durvalumab *vs* durvalumab+oleclumab *vs* durvalumab+monalizumab *vs* durvalumab+danvatirsen	Ⅰ; Ⅱ; Ⅲa	No	160	MPR	Feasibility to sugery; AEs; PCR
CheckMate 77T	NCT04025879	2019	Ⅲ	Chemotherapy+nivolumab *vs* chemotherapy+placebo	Ⅱ; Ⅲb	Uncertain	452	EFS	OS; PCR; MPR
IO+ (with radiation)									
	NCT03217071	2017	Ⅱ	Pembrolizumab *vs* pembrolizumab+SBRT	Ⅰ; Ⅱ; Ⅲa	6 mon	40	CD3^+^ T cells/ *μ*m^2^	OS; RFS; Distant metastases; AEs
	NCT02904954	2016	Ⅱ	Durvalumab *vs* durvalumab+SBRT	Ⅰ; Ⅱ; Ⅲa	1 year	60	DFS	ORR; PCR
	NCT02987998	2016	Ⅰ	Pembrolizumab+neoadjuvant concurrent chemoradiation+consolidation pembrolizumab	Ⅱa	Uncertain	9	Toxicity	PFS; ORR; PCR; OS; Nodal Downstaging
	NCT03237377	2017	Ⅱ	Durvalumab+radiation *vs* tremelimumab+radiation	Ⅲa; selected Ⅲb	Uncertain	32	Toxicities; Feasibility	Surgical morbidity and mortality; Path; RFS; OS
OS: overall survival; ORR: objective response rate; PFS: progression-free survival; DFS: disease-free survival; MPR: major pathologic responses; AEs: advent events; Path: pathology.									

## 3 新辅助免疫治疗的副反应

免疫检查点抑制剂取得巨大的临床获益及其作为一项肿瘤精准治疗方法，使人们容易忽视其引起的副反应。由于免疫检查点抑制剂在临床上的广泛应用，临床医生将越来越多地遇到免疫治疗相关副反应(immune-related adverse events, irAE)。因此，需要提高对这些副反应的临床表现，诊断和管理的认识^[27]^。研究发现，接受PD-1抑制剂治疗的NSCLC患者中，有7%-13%的患者出现3级或更高的毒性。在用PD-1和PD-L1抑制剂治疗的所有类型肿瘤患者中，高级别irAE的发生率低于20%^[28, 29]^。

虽然免疫治疗相关副反应发生率较低，但免疫检查点抑制剂有可能导致后果严重的副反应。*JAMA Oncol*上发表了迄今为止最大的一项免疫检查点抑制剂致死性副反应的评估性研究，来自7个中心的3, 545例数据显示接受免疫检查点抑制剂治疗的患者死亡率为0.6%^[30]^。尤其是对于新辅助治疗，严重的副反应可能给患者带来延迟性手术甚至致命性后果。尽管上述临床试验结果显示3级以上的副反应均较低，但副反应的发生某种意义上降低了获益人群，使后续临床试验的开展面临更多的挑战。在取得临床获益的同时最大限度减低副反应是其应用于临床须解决的问题。

## 4 新辅助免疫治疗面临的问题

### 4.1 新辅助免疫治疗受益人群的探索

如既往的新辅助化疗和放疗一样，术前新辅助免疫治疗并不能使所有治疗的人群都会获益。因此，目前新辅助免疫治疗所面临的最大的挑战仍是如何筛选出获益人群。寻找良好的标志物进行疗效预测。虽然目前尚无明确的疗效预测标志物，但经过Ⅲ期临床试验证实对免疫检查点抑制剂有指导意义的生物标志物主要有肿瘤PD-L1表达水平和肿瘤突变负荷(tumour mutation burden, TMB)，但其在肺癌中的应用仍有待进一步研究证实。尽管现有大部分临床试验显示肿瘤PD-L1表达较高时，检测点抑制剂可能有效，但这既不能保证此类药物用于PD-L1高表达的肿瘤一定有效，也不表示其对PD-L1表达阴性的肿瘤绝对无效。肿瘤在不同时间和空间以及经受不同的治疗方法时肿瘤细胞的PD-L1表达水平均有变化^[31]^。TMB在多项Ⅱ期临床试验中表现出一定的指导意义^[32]^，其相较肿瘤PD-L1表达水平的优势是可通过肿瘤组织和血液中ctDNA来监测。但检测方法上只能通过NGS、WES、靶基因测序来实现对肿瘤突变负荷的评估^[33]^。

CheckMate 159研究显示无论是否表达PD-L1患者均可获得主要病理缓解MPR。结果同时显示肿瘤突变负荷与患者达到MPR存在正相关。但LCMC3研究结果显示PD-L1阴性和PD-L1阳性患者中获得MPR的患者比例未达统计学差异；获得MPR和未获得MPR患者的TMB也无明显差异。NEOSTAR研究显示获得MPR的患者治疗前肿瘤PD-L1表达较高，且在PD-L1 > 1%的患者中治疗后残余肿瘤更少，进而得出治疗前PD-L1较高的患者可能获得更大的抗肿瘤效应。MK3475-223研究并未观察到PD-L1表达与MPR之间的相关关系。NCT02716038研究提示无论PD-L1表达水平，患者均可从新辅助免疫治疗中获益。

手术切除标本中淋巴结免疫细胞浸润与疗效间的关系尚在探索中，NEOSTAR研究中双药联合组新辅助免疫治疗后可诱导肿瘤局部CD3^+^ T细胞，T细胞多样性和记忆性T细胞的显著增加。LCMC3研究中获得MPR的患者出现NK细胞和粒细胞亚群的扩增、单核细胞亚群的减少。免疫治疗后肿瘤微环境中出现的免疫细胞改变可期待作为筛选受益人群的一种方法。

虽然不同研究的结果得出的结论并不一致，有些甚至相互矛盾；但预测免疫治疗疗效的标志物值得进一步研究。未来有可能纳入多种免疫反应指标(外周血免疫细胞亚群^[34]^、新的模型评估方法^[35, 36]^)来评估免疫状况和指导治疗及预测疗效。

### 4.2 新辅助治疗过程中疗效的评估

新辅助治疗面临又一大挑战是治疗过程中的疗效评估。免疫治疗发挥临床疗效持续的时间较长，应答模式也较多，主要有延迟反应、假性进展、混合缓解和超进展等。如何准确评估患者的临床状况并作出合理选择是一个值得研究的问题，在上述提到的研究中使用到的疗效评估方法主要是影像学评估和液体活检。

研究者们努力将影像学这一无创伤性检查作为对于免疫治疗疗效的评估。实体瘤疗效评价标准从2000年RECIST标准的发布到免疫治疗出现后到2014年欧洲内科肿瘤年会上研究者首次提出了实体肿瘤免疫相关疗效评价标准(immune-related Response Evaluation Criteria in Solid Tumors, irRECIST)^[37]^。irRECIST能否适用于新辅助免疫治疗中快速准确的疗效评估?这一问题值得进一步研究。为实现对免疫治疗反应的快速评估，分子影像学聚焦于检测肿瘤微环境中PD-L1表达，研究者开发了使用高特异性肽等新的显影剂通过PET检测肿瘤中PD-L1的表达水平旨在实现实时定量的检测^[38]^，以达到筛选人群和及时评估治疗反应的目的。但研究中常出现影像学与病理学结果相互矛盾的现象，影像学评估方法在免疫治疗中的指导作用有待于进一步探索。

液体活检是一种用于监测新辅助免疫治疗疗效的重要方法。只需进行简单的抽血，即可检测血浆中的循环肿瘤DNA (circulating tumor DNA, ctDNA)。因此可以根据治疗进程需要安全地重复进行，用来检测患者的疾病状况。在黑色素瘤相关研究领域显示ctDNA变化可以识别免疫治疗过程中出现的真性及假性进展^[39]^，进一步为临床治疗决策提供重要参考。CheckMate159研究结果显示ctDNA和外周血T细胞扩增可能作为筛选应答人群的方法^[34]^。但仍有许多亟待解决的关键性问题，诸如：如何准确筛选出ctDNA? ctDNA能否代表肿瘤细胞突变^[40]^?

PET-CT检查SUV在治疗前后的变化既往用于新辅助化疗/放疗的实体肿瘤疗效评估，在术前新辅助免疫治疗疗效评估中ChiCTR-OIC-17013726研究显示SUV下降超过30%可能作为一种新辅助免疫治疗后疗效评估的可靠方法。

### 4.3 新辅助免疫治疗临床研究的研究终点

以OS为主要研究终点的临床试验需要更多的人力物力投入。不同于新辅助化疗，多数新辅助免疫治疗将MPR作为观察终点。2014年*Lancet Oncol*专家共识提出MPR可以作为衡量新辅助化疗疗效的指标，且MPR与肺癌患者的远期预后相关，建议将MPR作为可切除肺癌新辅助治疗相关研究的替代终点。

目前对于病理学评估MPR上仍有争议。上述研究均采用将≤ 10%残留存活肿瘤定义为MPR。但因免疫治疗后病理反应模式与传统瘤床的评估有较大差异^[41]^，免疫检查点抑制剂产生的抗肿瘤效应导致肿瘤特异性T淋巴细胞等炎症细胞浸润，肿瘤细胞的坏死和水肿等非特异性病理表现增大了病理学评估的困难，因此部分学者提出了一种新的病理评估体系。该体系将退缩部分体积计入总瘤床，可更加精准地评估免疫治疗后病理反应^[42]^。

### 4.4 新辅助治疗手术介入的时机

新辅助免疫治疗过程中出现远处转移或疾病进展是患者需面临的风险。两项回顾性研究^[43, 44]^评估了可切除NSCLC延迟手术所需面临的风险。所以适时的手术介入是十分必要的。目前关于新辅助免疫治疗手术后时机选择的研究较少。上述研究选择在治疗结束后1周-3周后手术。因此我们期待正在进行的多项Ⅲ期临床研究来证实这一结果。

## 5 总结与展望

综上所述，虽然目前开展的小样本临床试验结果初步显示了新辅助免疫治疗是一种安全有效的NSCLC术前新辅助治疗手段，但同时也显示部分患者免疫治疗后可能会出现的严重副反应导致手术的延迟。此外新辅助免疫治疗也可能会增加手术切除的困难。因此这些问题值得进一步研究和探讨。目前临床试验中缺乏统一的疗效判断标准，尚需进一步探索能准确预测疗效的标志物以选择出更多的获益人群等。因此，新辅助免疫治疗在可切除NSCLC中的应用仍需要更多的临床研究和更多病例的经验积累以获得更可靠的临床证据才能逐步推广应用于临床^[45, 46]^。

## References

[1] Siegel RL, Miller KD, Jemal A (2019). Cancer statistics, 2019. CA Cancer J Clin.

[2] Zheng RS, Sun KX, Zhang SW (2019). Report of cancer epidemiology in China, 2015. Zhonghua Zhong Liu Za Zhi.

[3] Hui R, Özgüroğlu M, Villegas A (2019). Patient-reported outcomes with durvalumab after chemoradiotherapy in stage Ⅲ, unresectable non-small-cell lung cancer (PACIFIC): a randomised, controlled, phase 3 study. Lancet Oncol.

[4] Gettinger S, Horn L, Jackman D (2018). Five-year follow-up of nivolumab in previously treated advanced non-small-cell lung cancer: Results from the CA209-003 Study. J Clin Oncol.

[5] Ettinger DS, Wood DE, Aggarwal C (2019). NCCN Guidelines Insights: non-small cell lung cancer, version 1.2020. J Natl Compr Canc Netw.

[6] Perez-Gracia JL, Sanmamed MF, Melero I (2018). Neoadjuvant immunotherapy in non-small cell lung cancer: the sooner the better?. Transl Lung Cancer Res.

[7] Melero I, Berraondo P, Rodriguez-Ruiz ME (2016). Making the most of cancer surgery with neoadjuvant immunotherapy. Cancer Discov.

[8] Pardoll DM (2012). The blockade of immune checkpoints in cancer immunotherapy. Nat Rev Cancer.

[9] Keung EZ, Ukponmwan EU, Cogdill AP (2018). The rationale and emerging use of neoadjuvant immune checkpoint blockade for solid malignancies. Ann Surg Oncol.

[10] Liu J, Blake SJ, Yong MC (2016). Improved efficacy of neoadjuvant compared to adjuvant immunotherapy to eradicate metastatic disease. Cancer Discov.

[11] McGranahan N, Furness AJ, Rosenthal R (2016). Clonal neoantigens elicit T cell immunoreactivity and sensitivity to immune checkpoint blockade. Science.

[12] Markowitz GJ, Havel LS, Crowley MJ (2018). Immune reprogramming via PD-1 inhibition enhances early-stage lung cancer survival. JCI Insight.

[13] Forde PM, Chaft JE, Smith KN (2018). Neoadjuvant PD-1 blockade in resectable lung cancer. N Engl J Med.

[14] Doroshow DB, Sanmamed MF, Hastings K (2019). Immunotherapy in non-small cell lung cancer: facts and hopes. Clin Cancer Res.

[15] Bott MJ, Yang SC, Park BJ (2019). Initial results of pulmonary resection after neoadjuvant nivolumab in patients with resectable non-small cell lung cancer. J Thorac Cardiovasc Surg.

[16] Reuss JE, Smith KN, Anagnostou V (2019). Neoadjuvant nivolumab in resectable non-small cell lung cancer: Extended follow-up and molecular markers of response. J Clin Oncol.

[17] Cascone T, William WN, Weissferdt A (2018). Neoadjuvant nivolumab (N) or nivolumab plus ipilimumab (NI) for resectable non-small cell lung cancer (NSCLC). Ann Oncol.

[18] Cascone T, William WN, Weissferdt A (2019). Neoadjuvant nivolumab (N) or nivolumab plus ipilimumab (NI) for resectable non-small cell lung cancer (NSCLC): Clinical and correlative results from the NEOSTAR study. J Clin Oncol.

[19] Sepesi B, Cascone T, William W (2019). OA13.06 surgical outcomes following neoadjuvant nivolumab or nivolumab plus ipilimumab in non-small cell lung cancer - NEOSTAR study. J Thorac Oncol.

[20] Bar J, Urban D, Ofek E (2019). Neoadjuvant pembrolizumab (Pembro) for early stage non-small cell lung cancer (NSCLC): Updated report of a phase Ⅰ study, MK3475-223. J Clin Oncol.

[21] Li N, Ying J, Tao X (2019). P1.18-06 efficacy and safety of neoadjuvant PD-1 blockade with sintilimab in resectable non-small cell lung cancer. J Thorac Oncol.

[22] Li N, Ying J, Tao X (2019). Efficacy and safety of neoadjuvant PD-1 blockade with sintilimab in resectable squamous non-small cell lung cancer (sqNSCLC). J Clin Oncol.

[23] Gao S, Li N, Gao S (2020). Neoadjuvant PD-1 inhibitor (Sintilimab) in non-small cell lung cancer. J Thorac Oncol.

[24] Provencio M, Nadal E, Insa A (2019). Neoadjuvant chemo-immunotherapy for the treatment of stage ⅢA resectable non-small-cell lung cancer (NSCLC): A phase Ⅱ multicenter exploratory study-Final data of patients who underwent surgical assessment. J Clin Oncol.

[25] Zheng H, Zeltsman M, Zauderer MG (2017). Chemotherapy-induced immunomodulation in non-small-cell lung cancer: a rationale for combination chemoimmunotherapy. Immunotherapy.

[26] Shu CA, Grigg C, Chiuzan C (2018). Neoadjuvant atezolizumab + chemotherapy in resectable non-small cell lung cancer (NSCLC). J Clin Oncol.

[27] Martins F, Sofiya L, Sykiotis GP (2019). Adverse effects of immune-checkpoint inhibitors: epidemiology, management and surveillance. Nat Rev Clin Oncol.

[28] Puzanov I, Diab A, Abdallah K (2017). Managing toxicities associated with immune checkpoint inhibitors: consensus recommendations from the Society for Immunotherapy of Cancer (SITC) Toxicity Management Working Group. J Immunother Cancer.

[29] O'Kane GM, Labbé C, Doherty MK (2017). Monitoring and management of immune-related adverse events associated with programmed cell death protein-1 axis inhibitors in lung cancer. Oncologist.

[30] Wang DY, Salem JE, Cohen JV (2018). Fatal toxic effects associated with immune checkpoint inhibitors: a systematic review and meta -analysis. JAMA Oncol.

[31] Brody R, Zhang Y, Ballas M (2017). PD-L1 expression in advanced NSCLC: Insights into risk stratification and treatment selection from a systematic literature review. Lung Cancer.

[32] Goodman AM, Kato S, Bazhenova L (2017). Tumor mutational burden as an independent predictor of response to immunotherapy in diverse cancers. Mol Cancer Ther.

[33] Havel JJ, Chowell D, Chan TA (2019). The evolving landscape of biomarkers for checkpoint inhibitor immunotherapy. Nat Rev Cancer.

[34] Oezkan F, He K, Owen D (2018). MA04. 10 comprehensive peripheral blood immunophenotyping and T-cell clonal analysis during neoadjuvant immunotherapy with atezolizumab in NSCLC. J Thorac Oncol.

[35] Jafarnejad M, Gong C, Gabrielson E (2019). A computational model of neoadjuvant PD-1 inhibition in non-small cell lung cancer. AAPS J.

[36] Milberg O, Gong C, Jafarnejad M (2019). A QSP model for predicting clinical responses to monotherapy, combination and sequential therapy following CTLA-4, PD-1, and PD-L1 checkpoint blockade. Sci Rep.

[37] Tazdait M, Mezquita L, Lahmar J (2018). Patterns of responses in metastatic NSCLC during PD-1 or PDL-1 inhibitor therapy: Comparison of RECIST 1.1, irRECIST and iRECIST criteria. Eur J Cancer.

[38] Chatterjee S, Lesniak WG, Nimmagadda S (2017). Noninvasive imaging of immune checkpoint ligand PD-L1 in tumors and metastases for guiding immunotherapy. Mol Imag.

[39] Lee JH, Long GV, Menzies AM (2018). Association between circulating tumor dna and pseudoprogression in patients with metastatic melanoma treated with anti-programmed cell death 1 antibodies. JAMA Oncol.

[40] Razavi P, Li BT, Brown DN (2019). High-intensity sequencing reveals the sources of plasma circulating cell-free DNA variants. Nat Med.

[41] Sholl LM (2018). Understanding patterns of pathologic response following neoadjuvant immunotherapy for solid tumors. AnnOncol.

[42] Cottrell TR, Thompson ED, Forde PM (2018). Pathologic features of response to neoadjuvant anti-PD-1 in resected non-small-cell lung carcinoma: a proposal for quantitative immune-related pathologic response criteria (irPRC). Ann Oncol.

[43] Rice JD, Heidel J, Trivedi JR (2020). Optimal surgical timing after neoadjuvant therapy for stage Ⅲa non-small cell lung cancer. Ann Thorac Surg.

[44] Samson P, Patel A, Garrett T (2015). Effects of delayed surgical resection on short-term and long-term outcomes in clinical stage Ⅰ non-small cell lung cancer. Ann Thorac Surg.

[45] Karachaliou N, Fernandez-Bruno M, Bracht JWP (2018). Challenges and unanswered questions for the next decade of immune-oncology research in NSCLC. Transl Lung Cancer Res.

[46] Blumenthal GM, Bunn PA, Chaft JE (2018). Current status and future perspectives on neoadjuvant therapy in lung cancer. J Thorac Oncol.

